# Effect of *Laurus nobilis* L. Essential Oil and its Main Components on α-glucosidase and Reactive Oxygen Species Scavenging Activity

**Published:** 2013

**Authors:** Serap Sahin Basak, Ferda Candan

**Affiliations:** *Department of Biochemistry, Faculty of Sciences, Cumhuriyet University, 58140, Sivas, Turkey. *

**Keywords:** *α*-glucosidase, 1, 8-cineole, 1-(S)-*α*-pinene; Essential oil, *Laurus nobilis *L., R-(+)-limonene

## Abstract

The present study was designed to determine the effects of the essential oil of *Laurus nobilis *L. (Lauraceae) and its three main components on α-glucosidase and reactive oxygen species scavenging activity. The chemical composition of the essential oil from *Laurus nobilis *L. leaves was analyzed by GC/GC-MS and resulted in the identification of 29 compounds, representing 99.18% of the total oil. 1,8-cineole (68.82%), 1-(S)-*α*-pinene (6.94%), and R-(+)- limonene (3.04%) were determined to be the main components. The antioxidant features of the essential oil and its three main components were evaluated using inhibition of 2,2-diphenyl-1- picrylhydrazyl, hydroxyl, and superoxide radicals, inhibition of hydrogen peroxide and lipid peroxidation assays. The results show that the DPPH, hydroxyl, and superoxide radical as well as hydrogen peroxide scavenging activities of the essential oil are greater than the positive controls and the three main components of the oil when tested independently. The inhibition of lipid peroxidation by the oil occurred less frequently than with 1,8-cineole and R-(+)- limonene alone, but the effects were more pronounced than those seen with 1-(S)-*α*-pinene and the positive controls. An *α*-glucosidase inhibition assay was applied to evaluate the *in-vitro *antidiabetic activity of the essential oil. IC_50_-values were obtained for laurel essential oil, 1, 8-cineole, 1-(S)-*α*-pinene, and R-(+)-limonene: 1.748 μL/mL, 1.118 μL/mL, 1.420 μL/mL and 1.300 μL/mL, respectively. We also found that laurel essential oil and 1, 8-cineole inhibited the *α*-glucosidase competitively while 1-(S)-α-pinene and R-(+)-limonene were uncompetitive inhibitors.

## Introduction

Free radicals have one or more unpaired electrons and are highly reactive, short-lived compounds and have the ability to react with organic or inorganic molecules. The most significant free radicals in biological systems arise from oxygen. Aerobic organisms need oxygen to survive, but high concentrations of oxygen cause cellular damage (1). Antioxidant defense systems work to prevent damage from reactive oxygen species (ROS) such as hydrogen peroxide, singlet oxygen, and hydroxyl, peroxyl, and superoxide radicals. 

Diabetes (Diabetes mellitus (DM)) is a disease that is characterized by high blood glucose (hyperglycemia) due to the total or partial insulin deficiency. Long-term complications of uncontrolled high blood glucose levels include nephropathia, neuropathy, foot ulcers, gangrene, and coronary diseases (2). Although contemporary treatment for diabetes consists of insulin and oral antidiabetics, there is a growing tendency to search for new natural and synthetic antidiabetic drugs, especially in developing countries, due to the difficulty in obtaining and preserving current medicines in addition to undesired side effects of these drugs (3). One approach to decreasing postprandial blood glucose levels is to postpone glucose absorbtion by inhibiting α-amylase and α-glucosidase, which are digestive enzymes that hydrolyze carbohydrate (4). To this end, various pharmacological studies have emerged with the intent to discover new drugs targeted against α-amylase and *α*-glucosidase (4).

The role of reactive oxygen species in diabetes has been a widely discussed issue since the 1980›s, and there has recently been an increase in studies on the connection between oxidative stress, diabetes and diabetic complications (5). It has been observed that in diabetes, as a result of the increase in oxidative stress, the production of free radicals increases, but the production of antioxidants decreases. Thus, increased reactive oxygen species concentration is considered as one of the important complications of diabetes (6).

*Laurus nobilis *L. is a plant belonging to the Lauraceae family, which comprises approximately 2500 species. The genus Laurus is found in Europe and consists of the two species *Laurus azorica *and *Laurus nobilis*. The aforementioned tree grows between 3-10 m in height and possesses yellow flowers. Its leaves, which are not shed during winter, are 5-10 cm long, 2-5 cm wide, and green in color. The fruits are small and olive-like (7). For a long time now, essential oils obtained from plants have been used in various industries including medicine, food, and cosmetics; the oils exhibit beneficial functions such as antibacterial, antifungal, and antioxidant activities (8). The essential oil of *Laurus nobilis *L. is used in the production of soap and also as an aroma in the food and cosmetics industries; dry fruits and dry leaves are used for adding fragrance to food and consumed as tea, respectively (7). The antimicrobial, analgesic, anti-inflammatory, antitumoral, acetylcholine esterase inhibiting properties of the essential oil of *Laurus nobilis *L. have been reported (9-12).

Since it is known that reactive oxygen species induce diabetes, it was investigate that whether this essential oil can be effective in diabetes through the inhibition of *α*-glucosidase or by scavenging reactive oxygen species. As far as our literature survey could ascertain, the antioxidant activity of *Laurus nobilis *L. essential oil was reported only DPPH assay (13), but no information was available for *in-vitro α*-glucosidase inhibition activity. Hence, the aim of the present study was to examine the antioxidant properties of *Laurus nobilis *L. essential oil by using five different *in-vitro *methods, namely hydroxyl, superoxide, and DPPH radicals inhibition, hydrogen peroxide and lipid peroxidation inhibition activities. In addition, we also utilized an assay for *α*-glucosidase inhibition to evaluate *in-vitro *antidiabetic activity of the oil.

## Experimental

*Plant material*

*Laurus nobilis *L. leaves collected in June 2006 from Istanbul in Turkey were identified in the Biology Department of Cumhuriyet University by Dr. Erol DÖNMEZ. A voucher specimen ED (14251) SŞB was deposited in the Herbarium Laboratory in the Biology Department at the University of Cumhuriyet (CU).

*Extraction of the essential oil*

The leaves of *Laurus nobilis *L. were shade-dried and subjected to water distillation for 3 h using a Clevenger-type apparatus (yield 0.856 ± 0.018% v/w). The obtained essential oil was stored at 4°C until testing and analysis. All experiments were made at the same essential oil with three replications.

*Gas chromatography (GC)*

The GC analysis of the essential oil was performed using a Trace-GC-ultra with an INNOWAX capillary column (length 60 m, inner diameter and film thickness 0.25 mm 0.25 μm, respectively). Helium gas was used as the carrier at a flow rate of 1 mL/min. Injector and MS transfer line temperatures were set at 200 and 250ºC, respectively. The GC oven temperature was kept at 50ºC for 2 min, then increased to 250ºC at a rate of 5°C /min and held for 5 min. Diluted samples (1:100 v/v, in acetone) of 1.0 μL were injected manually in the splitless mode.

*Gas chromatography/Mass spectrometry (GC/MS)*

Analyses were conducted under the same column and conditions with GC interfaced with FINNIGAN Trace-DSQ mass spectrometer (ionization energy of 70 eV). The mass range was from an *m/z *of 50 to 450. Identification of compounds was based on comparisons of the relative retention indices and mass spectra with those of the WILEY and NIST library data standards of the GC/MS system.

*Antioxidative capacity*

*Hydroxyl radical scavenging activity*

Hydroxyl radical scavenging activity was carried out by measuring the hydroxyl radicals generated from the Fe^3+^/ascorbate/EDTA/H_2_O_2 _system (14). The attack of the hydroxyl radical to deoxyribose leads to the formation of thiobarbituric acid reactive substances (TBARS) (15). Hundred microlitre various concentrations of the samples (essential oil and three major components in *n*-hexane) were added to a reaction mixture containing 100 μL 3.0 mM deoxyribose, 100 μL 0.1 mM FeCl_3_, 100 μL 0.1 mM EDTA, 100 μL 0.1 mM ascorbic acid, 100 μL 1 mM H_2_O_2_, and 20 mM phosphate buffer (pH = 7.4), at a final volume of 1.0 mL. The reaction mixture was incubated at 37ºC for 1 h. Then, 1 mL of thiobarbituric acid (TBA, 1%) and 1.0 mL of trichloroacetic acid (TCA, 2.8%) were added to the test tubes and they were incubated at 100ºC for 20 min. After the mixtures being cooled, absorbance was measured at 532 nm against a blank containing deoxyribose and buffer. The percentage inhibition (I) of deoxyribose degradation was calculated in the following way:

% I = (A_0_ - A_1_ / A_0_) × 100

Here, A_0_ was the absorbance of the control reaction (containing all reagents except the test compound) and A_1_ was the absorbance of the test compound.

*Inhibition of superoxide radicals*

Superoxide radical generation by the xanthine/xanthine oxidase (EC 1.1.3.22) system was determined spectrophotometrically by monitoring the production of nitro blue tetrazolium (NBT) (16). 100 μL various concentrations of the samples(essential oil and three major components in *n-*hexane) were added to a reaction mixture containing 100 μL 2 nM xanthine, 100 μL 12 nM NBT, 100 μL 1.0 U/mL xanthine oxidase, and 0.1 M phosphate buffer (pH = 7.4), making up a final volume of 2.0 mL. After incubating the mixture at 25ºC for 10 min, the absorbance was read at 560 nm and compared with the control samples in which the enzyme was not included. The percent inhibition of superoxide anion was calculated using the following equation:

Inhibition% = (A_0_ - A_1_ / A_0_) × 100

In this equation, A_0_ was the absorbance of the control and A_1_ was the absorbance of the samples.

*Hydrogen peroxide scavenging activity*

The ability of the essential oil to scavenge hydrogen peroxide was determined spectrophotometrically as described previously (17). Briefly, a solution of hydrogen peroxide (2 mM) was prepared in 0.17 M phosphate buffer (pH = 7.4). 600 μL various concentrations of the samples (essential oil and three major components in *n-*hexane) were added to the reaction mixture containing 600 μL, 2 mM hydrogen peroxide. After 10 min of incubation at room temperature, the absorbance was read against a blank at 230 nm. The percentage hydrogen peroxide scavenging activity of samples was calculated as follows:

Scavenged% hydrogen peroxide = (A_0_ - A_1_ / A_0_) × 100

Here, A_0_ was the absorbance of the control and A_1_ was the absorbance of the presence of samples.

*Lipid peroxidation inhibition assay*

Assays for non-enzymatic lipid peroxidation were performed as described with minor changes (18). Rat liver (25% (w/v)) was homogenized with 40 mM Tris-HCl buffer (pH = 7.0) in three strokes. The homogenate was centrifuged at 10,000 g for 120 min and the supernatant was used in the experimental studies. A 1-mL sample of the reaction mixture contained 100 μL of different concentrations of the samples (essential oil and three major components in *n-*hexane), 100 μL supernatant, 20 μL of 1 mM FeCl_3_ and 20 μL of 1 mM ascorbic acid to induce hydroxyl radical generation. After an incubation period of 1 h at 37ºC, the extent of lipid peroxidation was measured by the TBA reaction. Then, 1 mL TBA and 1.0 mL 2.8% TCA were added and the test vials were heated to 100ºC for 20 min. After cooling, 2.5 mL *n*-butanol was added and the samples were centrifuged at 3500 rpm for 5 min. The absorbance was read at 532 nm. The percent inhibition of lipid peroxidation was calculated using the following equation:

Inhibition% = (A_0_ - A_1_ / A_0_) × 100

In this equation, A_0_ was the absorbance of the control and A_1_ was the absorbance of the samples.

*DPPH assay*

The DPPH assay was measured by following the bleaching of a purple methanol solution of DPPH (19). Hundred microlitre of various concentrations of the samples (essential oil and three major components in *n*-hexane) were added to 5 mL of a 0.004% solution of DPPH in methanol. After 30 min of incubation at room temperature, the absorbance was read against a blank at 517 nm. DPPH radical scavenging activity was calculated using the following formula:

Inhibition% of DPPH = (A_0_ - A_1_/A_0_) × 100

Here, A_0_ was the absorbance of the control and A_1_ was the absorbance of the presence of samples.

In all experiments curcumin, ascorbic acid and BHT were used as positive controls. They were dissolved in hexane (for hydroxyl, superoxide, lipid peroxidation assays (1 mg mL^-1^), DPPH assay (100 mg mL^-1^) and hydrogen peroxide assay (0.1 μg mL^-1^)) like essential oil and its three major components and added to the reaction mixture by micropipette with different volumes. Final concentration of these compounds calculated at μL mL^-1^ in reaction mixture and shown in that unit is displayed in Table 2. IC_50_-values (inhibitory concentration, 50%) were calculated from the dose-response curve (Sigma Plot Graph and statistical Program 9.0) obtained by plotting the percentage of inhibition versus the concentrations.

*Effect on α-glucosidase*

*α-glucosidase inhibitory assays*

A previously described bioassay method was used to measure *α*-glucosidase inhibition by samples (20). All reagents were pre-incubated for 15 min at 37ºC in a water bath. Then, 0.20 mL of the α-glucosidase (EC 3.2.1.20) enzyme solution (0.01 U in 50 mM sodium phosphate buffer with 100 mM NaCl and pH of 6.9) was added to 0.1 mL of increasing concentrations of samples and incubated for an additional 15 min at 37°C. Afterwards, 0.20 mL of the para-nitrophenol-*α*-D-glucopyranoside (pNPG) solution (2 mM pNPG in 50 mM sodium phosphate buffer with pH of 6.9) was added and incubated for 30 min at 37ºC, and the reaction was stopped by the addition of 1 mL 0.1 M Na_2_HPO_4_. The test tubes were cooled under tap water and the absorbance was measured at 400 nm. All reactions were carried out at 37ºC for 30 min with three replications.

Inhibitory activity of samples against *α*-glucosidase was calculated from the below equation:

Inhibition% = 100 - {[ (pNPG)_test_ / (pNPG)_control_ ] × 100}

**Table 1:** GC-MS Analysis of the isolated from *Laurus nobilis *L. leaves essential oil

**Compounds**	^a^ **RT**	^b^ **RI**	^c^ **%**
5,5-dimethylcyclopentadien	11.94	872	0.06
1-(S)-*α*–pinene	13.79	915	6.94
Camphene	15.27	945	1.01
2-*α*-pinene	16.61	972	1.70
Sabinene	16.86	977	2.10
l- phellandrene	18.42	1008	0.74
*α *– terpinene	18.85	1016	2.02
R-(+)-limonene	19.50	1028	3.04
1,8-cineole	19.82	1034	68.82
γ-terpinene	20.79	1052	1.61
(2-methylprop-1-enyl)-cyclohexa-1,3-diene	21.07	1057	0.14
Izomyrisenole	21.27	1061	0.02
p-cymene	21.64	1068	2.04
*α*-terpinolene	21.98	1074	0.83
5,9,9-trimethyl-spiro[3.5]non-5-en-1-on	22.26	1079	0.03
p-ment-1-en-3,8-diol	23.30	1099	0.04
3-hexane-1-ol	24.37	1119	0.06
Urea	26.24	1154	0.09
Benzene, 4-ethenyl-1,2-dimethyl	26.48	1159	0.11
*α*-campholen aldehyde	28.11	1189	0.03
L-linalool	28.57	1198	0.43
Endobornyl acetate	30.39	1234	0.71
Pinocarvone	30.57	1237	0.53
1,4-terpineole	30.73	1241	1.77
bicyclo[3.1.1]hep-2-en-2-carboxy aldehyde 6,6-dimethyl	31.84	1263	0.11
trans-pinocarveole	32.23	1270	0.98
2(1H)-naphthalenon-octahydro-8a-hidroksi-4a-methyl (cis)	32.32	1272	0.40
Geosmin	32.72	1280	0.22
p-ment-1-en-8-ol	32.90	1283	0.60
Total		99.18	

^a^ RT, Retention time (min).

^b ^RI,Retention index relative to (C_8_-C_16_) *n*-alkanes.

^ c^ %, Relative proportions of the essential oil components expressed as percentages obtained by GC-MS.

*Kinetic studies of α-glucosidase*

A calibration curve was generated using the pNPG standard (21). Briefly, 1 mL of 0.1 M Na_2_HPO_4_ solution was added to the increasing concentrations of pNPG solution (4.00 × 10^-4^ - 0.018 mg mL^-1^) and boiled for 10 min. The test tubes were cooled under tap water and the absorbance was measured at 400 nm.

*Control experiments*

All reagents were pre-incubated for 15 min at 37°C in a water bath. Then, 0.20 mL of *α*-glucosidase enzyme solution (0.01 U in 50 mM sodium phosphate buffer with 100 mM NaCl and pH of 6.9) and 0.10 mL buffer were added to 0.2 mL of increasing concentrations of pNPG solution [(0.125-2 mM) in 50 mM sodium phosphate buffer with pH of 6.9)]. After 3 min, the reaction was stopped by the adding 1 mL of 0.1 M Na_2_HPO_4_ and boiling for 10 min. The test tubes were then cooled under tap water and the absorbance was measured at 400 nm.

*Incorporation of the inhibitor*

A 0.10-mL sample of *Laurus nobilis *L. essential oil was incubated with 0.01 U of *α*-glucosidase enzyme solution in 0.20 mL for 15 min at 37°C. The above procedure was then repeated.

The Michaelis-Menten constant (Km) and maximal velocity (V_max_) in the presence and absence of essential oil of *Laurus nobilis *L. were determined using Lineweaver-Burk equations.

*Statistical analysis*

For the essential oil or standard compounds, three samples were prepared for the assay of each method. The data are presented as mean ± standard deviation of three determinations. Statistical analyses were performed using Sigma Plot graphs / statistical programme and Student’s t-test analysis. P-values < 0.01 were regarded at significant. IC_50_-values (inhibitory concentration, 50%) were calculated from the dose-response curve (Sigma Plot Graph and statistical Program 9.0) obtained by plotting the percentage of inhibition versus the concentrations.

## Results and Discussion

*Chemical composition of the essential oil*

Since the biological activity of an essential oil is dictated by the sum of its components, we first analyzed the chemical composition by GC-GC/MS of the oil isolated from *Laurus nobilis *L. leaves (Table 1). The analysis resulted in the identification of 29 compounds representing 99.18% of the total oil. The compounds 1,8-cineole (68.82%), 1-(S)- *α*-pinene (6.94%), and R-(+)-limonene (3.04%) were the major components, composing 78.80% of the oil. In previous studies, the main components of laurel essential oil were reported as: 1, 8-cineole, sabinene, *α*-pinene, linalool and *α*-terpinyl acetate (22-24). Dadalıoğlu and Evrendilek have stated that laurel essential oil has antibacterial properties and its main components are 1, 8-cineole, *α*-terpinene, and sabinene (25). Chemical compositions of essential oils mainly include monoterpenes, sesquiterpenes, and oxygenated derivatives. It was known that essential oils possess different biological effects, and these effects arise primarily from monoterpenic compounds (26).

The main component of laurel essential oil (1, 8-cineole) is found in the essential oils of many plants and is used in food as a sweetener, in aromatherapy as a skin stimulator, and in the treatment of bronchitis and asthma (27). Additionally, it is known that 1, 8-cineole has anti- inflammatory, antimicrobial, and antitumoral properties (28, 29). The anti-inflammatory and antibacterial properties of 1-(S)-*α*-pinene, which is the second most predominant component, have also been reported (30). The third most abundant compound of the laurel essential oil, R-(+)-limonene, is a monocyclic monoterpene used as an aroma in juices, drinks, puddings, and ice creams; it is also an ingredient in cosmetics, soaps, and many other cleaning products due to its pleasing aroma. The anti-inflammatory, antibacterial, antitumoral, antifungal, and anticarcinogenic activities of R-(+)-limonene have also been reported (26, 30-32).

*Antioxidant activity*

**Figure 1:**
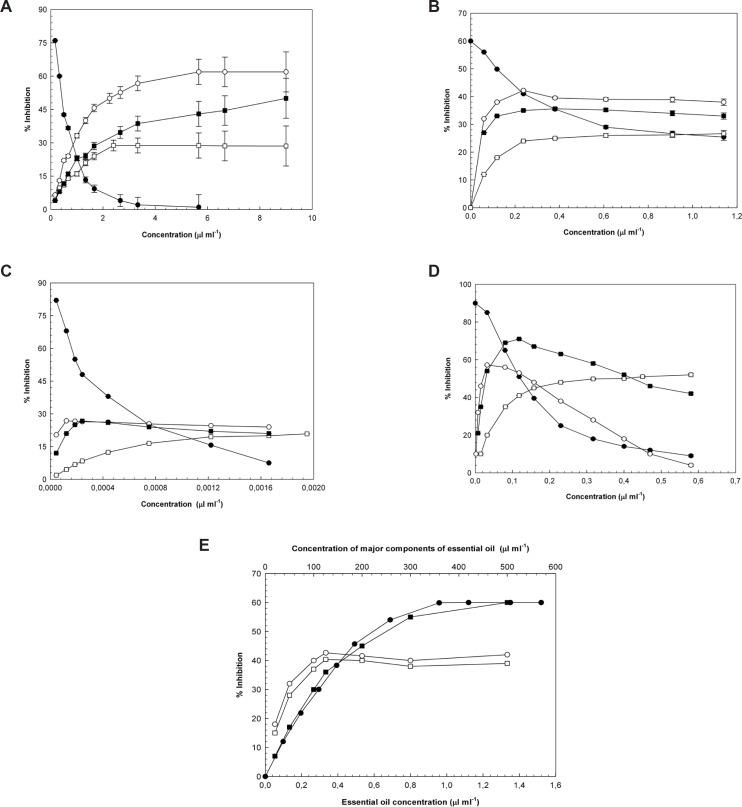
a: Hydroxyl radical inhibition activity of (●) essential oil (○) 1,8-cineole (□) 1-(S)-α-pinene, and (■) R-(+)-limonene. The results were expressed as the mean ± SEM; n = 3. b: Superoxide radical inhibition activity of (●) essential oil (○) 1,8-cineole (□) 1-(S)- *α*-pinene, and (■) R-(+)-limonene. The results were expressed as the mean ± SEM; n = 3. c: Hydrogen peroxide inhibition activity of (●) essential oil (○) 1,8-cineole (□) 1-(S)-*α*-pinene, and (■) R-(+)-limonene. The results were expressed as the mean ± SEM; n = 3. d: Lipid peroxidation inhibition activity of (●) essential oil (○) 1,8-cineole (□) 1-(S)-*α*-pinene, and (■) R-(+)-limonene. The results were expressed as the mean ± SEM; n = 3. e: DPPH radical inhibition activity of (●) essential oil (○) 1,8-cineole (□) 1-(S)-α-pinene, and (■) R-(+)-limonene. The results were expressed as the mean ± SEM; n = 3.

Since 1980, the role of reactive species in diabetes has been widely debated, which has led to a greater effort to describe a connection between oxidative stress, diabetes, and diabetic complications. Increased oxidative stress in diabetes, results in increased free radical formation. At the same time, it appears that the antioxidant production decreases diabetes; therefore, it is now accepted that an increased free radical level is an important complication of diabetes (33). The effects of essential oil and its main components on DPPH, hydroxyl and superoxide radicals, hydrogen peroxide levels, and lipid peroxidation are shown in Figure 1. The IC_50_-values (inhibitory concentration, 50%) for the essential oil of *Laurus nobilis *L. leaves, its main components, and positive controls for the inhibition of hydroxyl and superoxide radicals, hydrogen peroxide, lipid peroxidation and DPPH were illustrated in Table 2.

**Table 2:** Antioxidant activity of the isolated from *Laurus nobilis *L. leaves essential oil, its main components and positive standarts.

**Sample**	**Hydroxyl** ^a^ **IC**_50_ ** (μL/mL)**	**Süperoxide** ^a^ **IC**_50_ ** (μL/mL)**	**Hydrogene peroxide** ^a^ **IC**_50_ **x10** ^4^ **(μL/mL)**	**Lipid peroxidation** ^a^ **IC**_50_ ** (μL/mL)**	**DPPH** **IC**_50_ ** (μL/mL)**
Essential oil	0.398 ± 0.028	0.141 ± 0.004	2.421 ± 0.136	0.124 ± 0.003	0.575 ± 0.060
Curcumin^b^	13.56 ± 1.190	8.655 ± 0.222	3.70 ± 0.10	1.220 ± 2.013	9.60 ± 0.46
Ascorbic acid^b^	nt	92.450 ± 4.14	11.20 ± 0.56	nt	17.57 ± 2.86
BHT^b ^	32.00 ± 1.61	62.301 ± 0.903	6.984 ± 0.337	3.022 ± 1.346	23.750 ±0.500
1,8 cineole	2.25 ±0.12	-	-	0.020 ± 0.221	-
1-(S)-*α*–pinene	-	-	-	0.328 ± 0.780	-
R-(+)-limonene	8.95 ± 0.34	-	-	0.024 ± 0.580	283.62 ± 2.87

^a^Values are expressed as mean ± SD three different experiments.

^b^ Curcumin, ascorbic acid and BHT were used as positive controls.

nt: not tested

Hydroxyl radicals generated by the reduction of hydrogen peroxide and radiation, contribute significantly to the molecular and cellular damage within biological systems. Thus, scavenging and preventing the formation of hydroxyl radicals is of upmost importance. It was determined that the essential oil of laurel is capable of scavenging hydroxyl radicals (·OH) generated by an *in-vitro *Fe^3+^-ascorbate-EDTA-H_2_O_2_ system. According to the extrapolated IC_50_-values, laurel essential oil exhibits the highest inhibitory activity against hydroxyl radicals. Curcumin, which was one of the positive controls, is a more potent hydroxyl radical inhibitor than BHT. Since ascorbic acid was present in the experimental medium, its hydroxyl radical inhibitory activity was not studied. The presented IC_50_-values also demonstrate that 1, 8-cineole is a better hydroxyl radical scavenger than R-(+)-limonene. Even at its greatest concentration (2.42 μL/mL), 1-(S)-*α*-pinene only inhibited hydroxyl radical formation by 28.78%.

Superoxide radicals are produced from normal cellular functions, and they serve as catalysts for the formation of other various radical species. Because of its direct ties to different diseases, inhibition of superoxide becomes more important. Of all the samples tested, laurel essential oil, which has the lowest IC_50_-value, exhibits the strongest antioxidant activity against superoxide. Superoxide radical scavenging activities of the positive controls increased curcumin, BHT, and ascorbic acid, respectively. IC_50_-values for 1, 8-cineole, 1-(S)-*α*-pinene, and R-(+)-limonene were not determined. The highest superoxide radical inhibition percentages achieved for 1, 8-cineole (0.238 μL/mL), 1-(S)-*α*-pinene (1.136 μL/mL), and R-(+)-limonene (0.380 μL/mL) were 42.20%, 26.67%, and 35.62%, respectively.

Hydrogen peroxide is not reactive on its own, but, under appropriate conditions, it can form hydroxyl radicals, which are the most reactive oxygen radical species. When the hydrogen peroxide inhibition activities of the samples were measured, it was observed that laurel essential oil is the most active. Among the positive controls, ascorbic acid showed the most activity, followed by BHT and then curcumin. Similar to what was seen for superoxide scavenging activity, 1,8-cineole, 1-(S)-*α*-pinene, and R-(+)-limonene did not achieve 50% inhibition of hydrogen peroxide levels; their respective inhibition percentages were measured to be 26.81% (1.22 × 10^-4^ μL/mL), 20.85% (19.5 × 10^-^4 μL/mL), and 26.74% (2.43 × 10^-4^ μL/mL).

It has been reported that plasma lipoproteins, erythrocyte membrane lipids, and various tissue lipid peroxidation levels were elevated in diabetic patients (34). In addition, many studies have elucidated a link between diabetic complications and lipid peroxidation. In the results presented here, laurel essential oil exhibited greater activity than the positive controls against lipid peroxidation. Among the positive controls, curcumin was more effective at inhibiting lipid peroxidation than BHT. Since ascorbic acid was present in the experimental medium, its potential to inhibit lipid peroxidation was not evaluated. The IC_50_-values for inhibition of lipid peroxidation were obtained for (in order of least to greatest) 1-(S)-*α*-pinene, R-(+)-limonene, and 1,8-cineole.

If antioxidants are present in the test environment, DPPH undergoes a characteristic color change from violet to colorless. According to this colorimetric assay, the DPPH radical scavenging activity of the essential oil was higher than the activity of the positive controls. The DPPH radical scavenging activity of the positive controls sequentially decreased for curcumin, ascorbic acid, and BHT. Although the IC_50_-value for R-(+)-limonene could be extrapolated, IC_50_-values for 1, 8-cineole and 1-(S)-*α*-pinene were not determined; the highest activities measured were 42.65% (125 μL/mL) and 40.37% (125 μL/mL), respectively.

*Effect on α-glucosidase*

In cultures the world over, different plants are used for the treatment of diabetes, especially in traditional Chinese and Indian medicine (3). Diabetes is a worldwide illness leading to many disease complications. Control of postprandial blood glucose levels is first in the prevention of such complications, hence the current focus on developing inhibitors of α-amylase and *α*-glucosidase. Since the inhibitors of α-glucosidase decrease glucose absorption rates and depress the postprandial blood glucose levels, compounds of this type have become quite important for controlling Type II diabetes (4). Whereas, antidiabetic activities of herbal extracts have been widely studied and discussed, rarely to meet antidiabetic activity of essential oil (35-36). It was determined that the essential oil of *Laurus nobilis *L. inhibits the α-amylase by competitive mechanism (36). The inhibitory activity of laurel essential oil and its main components against *α*-glucosidase were reported in Table 3.

**Table 3:** Inhibitory activity of laurel essential oil and its three main components againts *α*-glucosidase.

**Sample**	**Concentration** **(μl/mL)**	**% İnhibition** ^a^
Essential oil		
	0.606	20.29 ± 0.18
	1.212	38.80 ± 0.78
	1.828	54.14 ± 0.65
	2.600	65.32 ± 0.45
	3.636	77.06 ± 1.25
	4.700	84.25 ± 0.96
	6.060	89.10 ± 0.28
	7.500	92.85 ± 1.01
1,8 cineole	1.118	50.00 ± 0.97
1-(S)-*α*–pinene	1.420	50.00 ± 0.65
R-(+)-limonene	1.300	50.00 ± 0.32

^a^ all determinations were carried out in triplicate and averaged. the α-glucosidase inhibitory activity (%) was defined as the percent decrease in the pNPG rate over the control

Laurel essential oil was found to inhibit α-glucosidase over 90%. The IC_50_-value of the oil was determined to be 1.748 ± 0.021 μL/mL. IC_50_-values of major components were increased in 1,8-cineole, R-(+)-limonene and 1-(S)-*α*-pinene, respectively. As the high IC_50_-values indicated the low inhibition activity, *α*-glucosidase inhibition activity of major components of essential oil were increased in 1-(S)-*α*-pinene, R-(+)-limonene and 1,8-cineole. On the other hand, total *α*-glucosidase inhibition percentages and concentration for individual components were as follows: 1,8-cineole (82.20%, 3.489 μL/mL), 1-(S)-*α*-pinene (60.02%, 1.815 μL/mL), and R-(+)-limonene (70.25%, 2.633 μL/mL).

Although the amounts of these compounds (terpenic compounds) within the oils are extremely small, their possible synergistic and antagonistic effects have to be taken into consideration. Since the essential oils are complex and impure mixtures, their biological activities vary in accordance with the compounds that they include; while there can be a more powerful biological activity as a result of the synergistic effect that is formed by the compounds coming together, sometimes the antagonistic effect leading to lower activity can also be observed (37,38). While essential oil concentration is increased, reactive oxygen scavenging activities of oil is decreased as shown in Figure 1(a), (b), (c) and (d). Although amount of essential oil increase in reaction medium, antagonistic property of oil component to predominate and decreased the reactive oxygen scavenging activity of essential oil according to the main components of the oil. Conversely in Figure 1 (e), together with amount of essential oil increase in reaction medium, synergistic property of oil component to predominate and increased the reactive oxygen scavenging activity of essential oil as regards the main components of the oil.

*Kinetic studies*

**Figure 2:**
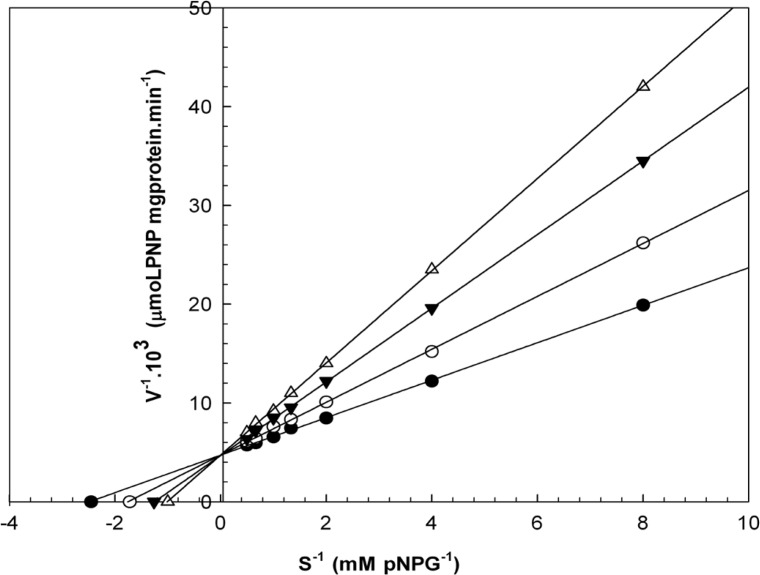
Lineweaver-Burk plots derived from the inhibition of α-glucosidase by essential oil. α-glucosidase was treated with each stated concentration of pNPG (0.125-2 mM) in the absence and presence of essential oil. The concentrations of essential oil were: (●) no inhibitor; (○) 0.458 μL/mL; (▼) 0.950 μL/mL; and (Δ) 1.830 μL/mL. The enzyme reaction was performed by incubating the mixture at 37ºC for 30 min.

**Figure 3:**
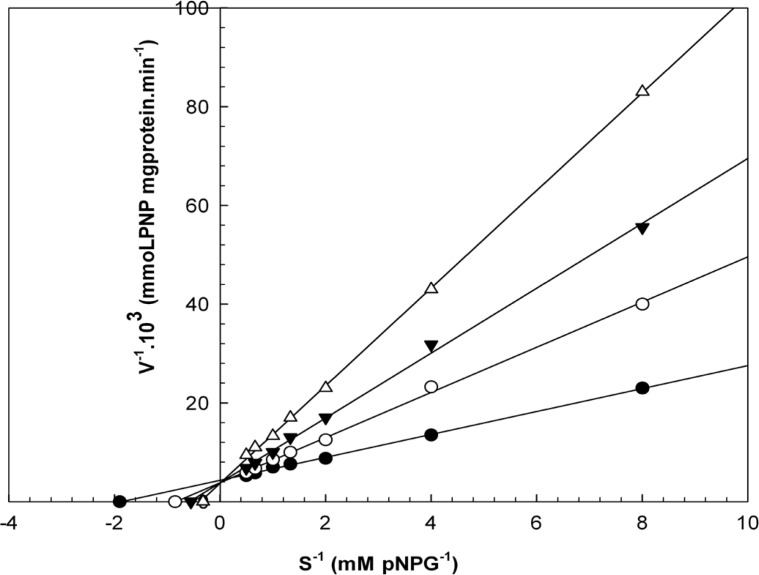
Lineweaver-Burk plots derived from the inhibition of α-glucosidase by 1,8-cineole. α-glucosidase was treated with each stated concentration of pNPG (0.1252- mM) in the absence and presence of 1,8-cineole. The concentrations of 1,8-cineole were: (●) no inhibitor; (○) 0.130 μL/mL; (▼) 0.520 μL/mL; and (Δ) 1.260 μL/mL. The enzyme reaction was performed by incubating the mixture at 37ºC for 30 min.

**Figure 4:**
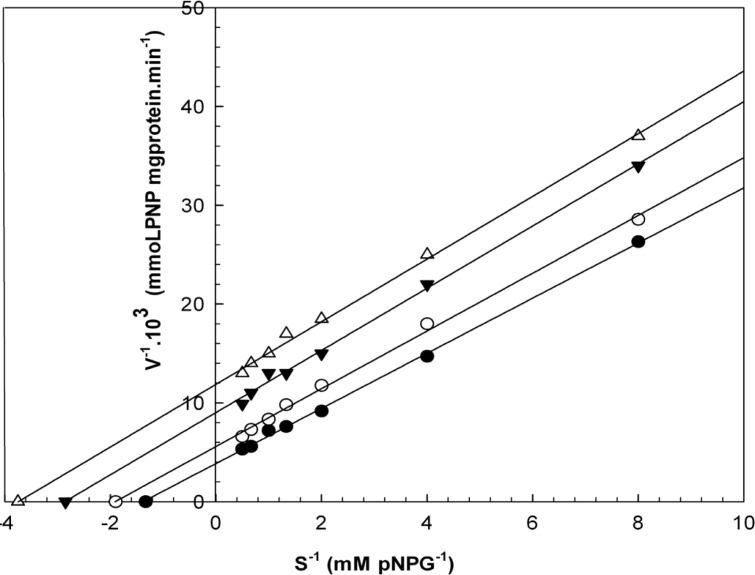
Lineweaver-Burk plots derived from the inhibition of α-glucosidase by 1-(S)-α-pinene. α-glucosidase was treated with each stated concentration of pNPG (0.125-2 mM) in the absence and presence of 1-(S)-α-pinene. The concentrations of 1-(S)-α-pinene were: (●) no inhibitor; (○) 0.250 μL/mL; (▼) 0.580 μL/mL; and (Δ) 1.160 μL/mL. The enzyme reaction was performed by incubating the mixture at 37ºC for 30 min.

**Figure 5:**
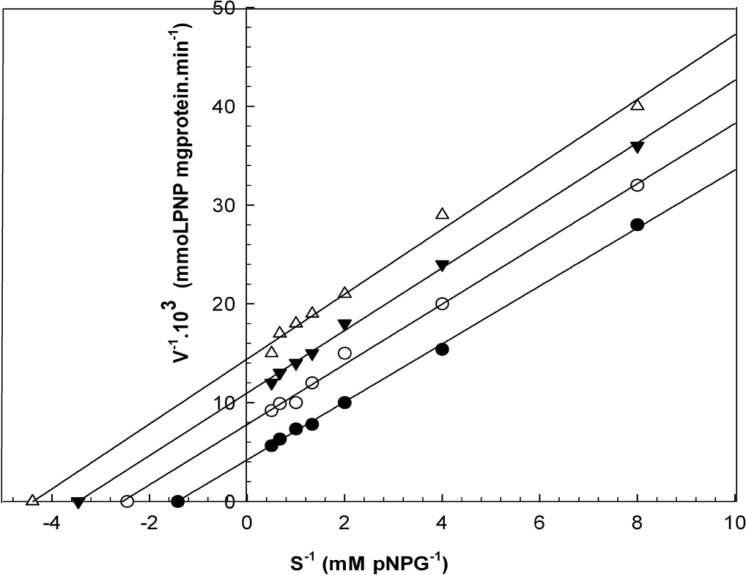
Lineweaver-Burk plots derived from the inhibition of α-glucosidase by R-(+)-limonene. α-glucosidase was treated with each stated concentration of pNPG (0.125-2 mM) in the absence and presence of R-(+)-limonene. The concentrations of R-(+)-limonene were: (●) no inhibitor; (○) 0.290 μL/mL; (▼) 0.582 μL/mL; and (Δ) 1.180 μL/mL. The enzyme reaction was performed by incubating the mixture at 37ºC for 30 min.

When varying concentrations of laurel essential oil and 1,8-cineole were analyzed, the maximal velocities (V_max_, y-intercept remained) did not change. However, values for the Michaelis-Menten constant (K_m_, slope of the trend lines) was increased (Figures 2 and 3) for these competitive inhibitors. The inhibitors are competing with substrate for the active site of the free enzyme and cause inhibition by forming an enzyme-inhibitor complex. For this reason, the inhibition can be prevented by increasing the concentration of substrate. The K_m_-values for laurel essential oil and 1,8-cineole were plotted by the least squares method against concentrations that demonstrated competitive inhibition to give inhibitory constants (K_i_) of 1.53 ± 0.71 μL/mL and 0.55 ± 0.27 μL/mL, respectively. In contrast, varying concentrations of 1-(S)- *α*-pinene and R-(+)-limonene resulted in a decrease in both maximal velocity and Michaelis-Menten constant values (Figures 4 and 5). This result suggests that 1-(S)-*α*-pinene and R-(+)-limonene inhibit α-glucosidase via an uncompetitive mechanism. When the concentrations of 1-(S)-*α*-pinene and R-(+)- limonene were plotted against 1/V_max(observed)_, the K_i_ was determined to be 0.70 ± 0.05 μL/mL and 0.20 ± 0.03 μL/mL, respectively, via the least squares method. 

## Conclusion

We have provided the first detailed study of the effects of the essential oil of *Laurus nobilis *L. and its three major components on *α*-glucosidase and *in-vitro *antioxidant activity. We were supply innovation that *α*-glucosidase inhibition activity of laurel essential oil and its three main components which were considered have many biological activities. The results of this study indicate that the essential oil and 1,8-cineole inhibit *α*-glucosidase by competitive inhibition, but 1-(S)-*α*-pinene and R-(+)-limonene are uncompetitive inhibitors. It was found that essential oil of *Laurus nobilis *L. and its three major components possess an *in-vitro *antioxidant property, thus inhibiting reactive oxygen species (ROS) such as hydroxyl and superoxide radicals, hydrogen peroxide (which is not a free radical but a reactive oxygen type), lipid peroxidation, and 2,2,diphenylpicrylhydrazyl (DPPH), which is a stable free radical. Since the results suggest that the essential oil obtained from laurel leaves and its main components inhibit α-glucosidase, the whole oil or its main components could be effective in the treatment of diabetes by scavenging reactive oxygen species and inhibiting α-glucosidase. However, these results need to be confirmed by *in-vivo *experiments.

*Author disclosure statement*

No competing financial interests exist.
